# Philanthropy as a Career

**Published:** 1887-07-16

**Authors:** 


### PHILANTHROPY AS A CAREER.

There are probably many people in Great Britain at the present time who have wealth, education, and leisure, whose life is something of a weariness to them because they have nothing very definite to do. Money is a good thing, and leisure is a good thing, but the man who is compelled to work for a livelihood probably gets much more satisfaction out of life than he who is not so compelled. Many a man becomes famous who would never have been heard of if he had been born rich, and many a man who might have become a great statesman, soldier, lawyer, divine, or philanthropist, is dawdling away his time and brains in obscurity, because somebody was ill-advised enough to leave him a small independency. The writer knows a man who spends fifty pounds a year in tobacco and cigars, mostly for his own use, because his father left him twelve or fourteen hundred a year, and gave him no occupation.

It is not our business to teach religion, but experience shows that man is not of the same order of being as the wild or tame animals, which have no instincts beyond the satisfaction of their immediate desires. Account for it as we will, it is as certain as the seasons that man is a being who looks before and after, and who has in his very constitution that which makes him feel and acknowledge a personal responsibility to something outside himself. So that most well-conditioned and intelligent persons who spend a purposeless life will admit that they ought not to spend life after such a fashion; and many will even go further, and say that they would do something purpose-like and useful for the world, if only they knew exactly how to set about it. There are a good many men in this condition, and perhaps a still larger number of women.

It is disappointing to a man who is able to set himself to work to find in the course of his journey through life so many intelligent and well-meaning human beings who cannot do so. Given a certain amount of education, leisure, courage, and intelligence, one would think that no man or woman who was really anxious about the matter need wait very long for a suitable career. Some very appropriate lines of Tennyson's here occur to the mind, but as this is intended to be a practical article, which it is hoped may make some converts, we will avoid the mere literary prettiness which the poetry might give, and confine ourselves to simple and direct arguments.

Politics, the Church, the army, the law, medicine, and commerce, offer each a distinct career to men of intellect and force of character. Such men require a career of some kind. A Bismarck, a Salisbury, a Gladstone, an Arnold, and numbers of others who might be named, could no more sit down at home smoking away their time in inglorious ease than they could play at tennis all day like a curate, or spend their days in a round of calls and receptions like a lady of fashion. Certain men, whatever wealth they are born to, must and will have a career, and these men never fail to find one.

All men cannot be politicians. Many cannot be clergymen or soldiers. Few persons of a fine sense of honour seek the prizes of the bar except under the pressure of necessity. Physic has little attraction for men of highly sensitive natures. Commerce, unless a man be bred to it and have the money-making instinct strong within him, had better be avoided. Philanthropy as a career, as the daily and serious business of a lifetime, is seldom even thought of. Happily, the pathway does not lead altogether through trackless forests. One illustrious pioneer has been before us and left a name which all men honour wherever civilisation has extended her empire. We do not assert that the late Lord Shaftesbury was the only philanthropist of modern times, but he, more than most others, made philanthropy a business, a real and earnest daily work, which he performed with the same regularity, diligence, and business-like capacity as the builder who erects houses, or the grocer who lives by selling sugar and tea.

Suppose a man wishes to become famous, what nobler name could he hand down to posterity than a name like that of Lord Shaftesbury? If he will think for a moment, there are few politicians or soldiers who will longer be remembered, and none whose name will be pronounced with greater reverence by generations of men yet unborn.

Probably, however, no one will ever be a great philanthropist who has an eager desire for fame. It is questionable whether a man can be great at all who has ardent longings in that direction. He who courts publicity, and cannot be happy unless his name is in everybody's mouth, is very near akin to him who enters the army because of the gay uniform, or who buys the yellowest of kid gloves and the most brilliant of neckties in order that everybody may notice him as he passes in and out of church. The true philanthropist is made of better stuff. He must be a man of sterling mental and moral gold, or he will not long wear the stamp of this most royal of all mints. But love of notoriety is not a very marked characteristic of Englishmen. It is not our way to strike an attitude, and call upon all the world to attend when we are going to do anything good. Many of our countrymen and country-women are in the fullest sympathy with that passage in the New Testament—" When thou doest alms, let not thy left hand know what thy right hand doeth." And we are strongly of opinion that there are a great number of people of both sexes in various parts of the country who would gladly come forward without a single thought of gain or fame, and lead or follow in an army of philanthropists, if they did but know the way. There is great and noble work for such an army of philanthropists to do, if they could only set themselves to it. Hospitals above all need men and women even more than they need money. Is there not a man of means and leisure anywhere in England who feels the fire of a noble charity burning within him, who will give himself now to duties so truly great? The Church has had her heroes, both men and women, young, middle-aged, and old. Cannot anyone be found who, from pure love of man, of man for his own sake and of man for God's sake, will offer himself wholly and entirely to this work—this and no other? Let no one think we are writing for writing's sake. Whoever takes that view of the subject must take it if it so pleases him. The facts remain that in the field of philanthropy there is work of the most profoundly important and urgent kind to be done, and there are few or no leaders who give themselves up to this and nothing else. In our judgment no career could be greater or more worthy of the attention of the most generous spirits and the ablest minds. If any readers desire to try philanthropy as a career, and will send us their names and addresses in confidence, we shall be glad to show them how they can set themselves practically to work without delay or difficulty.

